# Successful Removal of Giant Intrapericardial Paraganglioma via Posterolateral Thoracotomy

**DOI:** 10.1155/2014/308462

**Published:** 2014-11-09

**Authors:** Yoko Yamamoto, Ken Kodama, Hiroyuki Yamato, Masashi Takeda

**Affiliations:** ^1^Department of Thoracic Surgery, Yao Municipal Hospital, 1-3-1 Ryuge-cho, Yao, Osaka 581-0069, Japan; ^2^Department of Pathology, Yao Municipal Hospital, 1-3-1 Ryuge-cho, Yao, Osaka 581-0069, Japan

## Abstract

Intrapericardial paraganglioma remains a surgical challenge because of its hypervascular nature and firm adhesion to adjacent mediastinal structures. Here, we describe a 63-year-old female with a giant nonfunctioning intrapericardial paraganglioma tightly adhered to the left atrium. Marginal but complete resection of the tumor was achieved via right posterolateral thoracotomy. At the time of dissection between the tumor and the left atrial wall, we encountered massive hemorrhage leading to cardiac arrest. We were able to repair the wall laceration with minimal time under an optimal operative field, which avoids air embolism. She was discharged without complications and is currently in good health with no recurrence or metastasis for 15 months. Based on our experience, cardiopulmonary bypass should be considered, if surgeons are able to secure suitable sites for arterial and venous cannulations while right posterolateral thoracotomy is employed.

## 1. Introduction

Paraganglioma of the mediastinum is extremely rare and difficult to remove because of its hypervascular characteristics and the fact that many of them directly invade or firmly adhere to adjacent structures such as the heart, great vessels, trachea, esophagus, and spine. Complete resection is the only potential curative treatment [1]. We present a case of nonfunctioning intrapericardial paraganglioma that was successfully removed through right posterolateral thoracotomy.

## 2. Case

**Figure 1:**
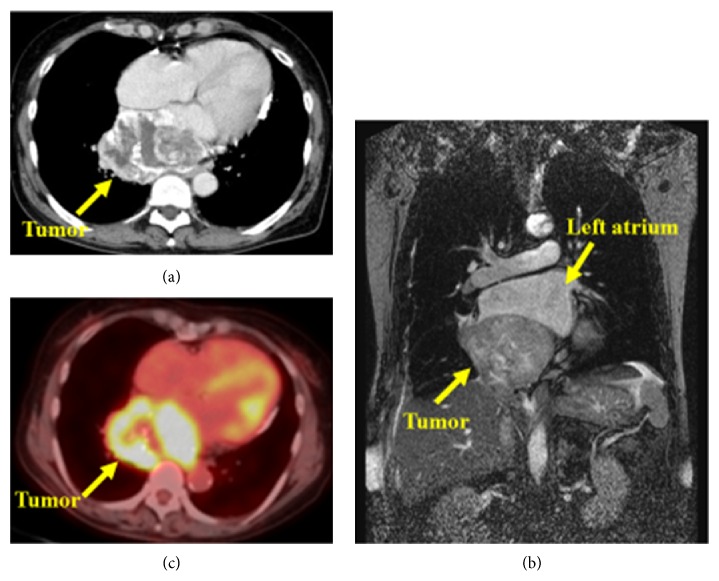
(a) Dynamic CT shows the mass 65 × 90 mm in diameter in the middle mediastinum which is hyperdynamic enhancement continuing from the early to delayed phase. (b) MRI of the chest demonstrating a large tumor compressing the left atrium. The tumor and left atrium are indicated with arrows. (c) PET-CT image showing a mediastinum mass with the SUVmax of 10.5.

**Figure 2:**
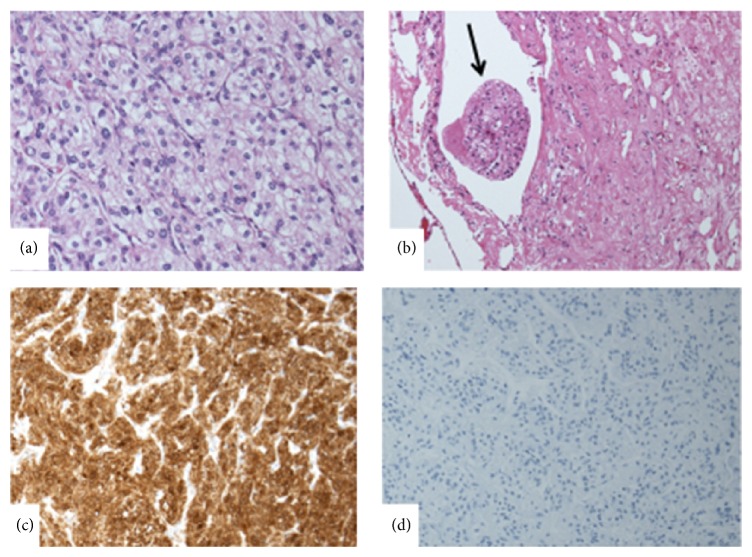
Paraganglioma showing a nesting (Zellballen) pattern (a) with vascular-rich stroma and vascular invasion (b) (hematoxylin and eosin). Immunohistochemistry results showing presence of NSE (c) and absence of S100 protein (d) markers.

A 63-year-old female had been suffering from dysphagia and was referred to our hospital after computed tomography (CT) scanning revealed a large middle mediastinal mass. Her medical history was unremarkable. Physical examination revealed normal vital signs at the time of admission. Laboratory findings including tumor markers were within normal limits. Chest CT scan revealed a well-demarcated mass 90 × 65 mm in diameter in the middle mediastinum. Dynamic CT scan showed hyperdynamic enhancement continuing from the early to delayed phase (Figure 1(a)). The degree of enhancement was similar to that of vascular structures. There were calcifications in the tumor. The tumor did not seem to be associated with invasion to the surrounding structures. Magnetic resonance imaging (MRI) showed an isointense tumor with muscle on the T1-weighted image and high intensity on the T2-weighted image. MRI demonstrated a large tumor with tight compression of the left atrium and the right inferior pulmonary vein, and the tumor extended into the hilum of the right lower lobe (Figure 1(b)). Positron emission tomography (PET) revealed high uptake of F-18 fluorodeoxyglucose (FDG) with a maximal standardized uptake value (SUVmax) of 10.5 (Figure 1(c)). As a malignant mesenchymal tumor such as high-grade sarcoma or lymphoma was suspected, we performed right-sided video assisted thoracic surgery (VATS) to obtain tissue biopsies. During the procedure, the tumor was hemorrhagic. We were able to control the bleeding and obtain biopsy material. Microscopically, the mass was composed of cells arranged in a nesting pattern (Zellballen) (Figure 2(a)) with vascular-rich stroma and vascular invasion (Figure 2(b)). Tumor cells were strongly immunoreactive to neuron specific enolase (NSE) (Figure 2(c)) and CD56 but negative for chromogranin A, synaptophysin, and S100 protein (Figure 2(d)). The Ki-67 labeling index was 7.5%. Sequence analysis showed no fusion genes that define any type of sarcoma. In addition, her blood pressure was stable and within normal limits perioperatively. These findings supported a diagnosis of nonfunctioning paraganglioma.

**Figure 3:**
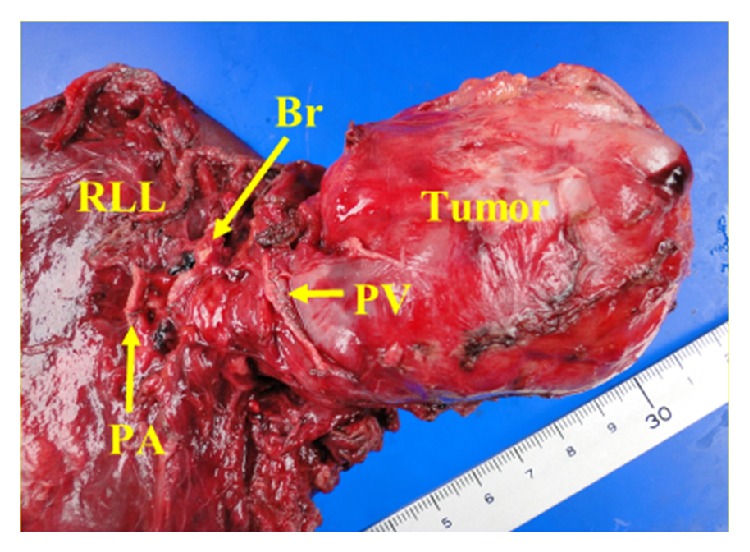
Resected specimen. The tumor was marginally but completely resected with right lower lobe of the lung. RLL: right lower lobe; Br: lower lobe bronchus; PA: pulmonary artery; PV: pulmonary vein.

On the basis of these imaging and pathologic findings, we planned complete resection as the first choice of treatment for this patient. A posterolateral thoracotomy was performed under general anesthesia with one-lung ventilation. We could palpate the huge tumor in the pericardium. The tumor directly extended into the right lower lobe of the lung beneath the inferior pulmonary vein, which had been markedly shifted cranially by the mass effect. The pericardium bulge due to the tumor was opened longitudinally behind the right phrenic nerve. However, we could not identify the border between tumor and left atrial wall by digital manipulation. Therefore, we employed ultrasound examination. As a result, we were able to identify the border and recognized that there was no tumor invasion to the left atrial wall. Thus, we decided to perform en bloc resection of paraganglioma with right lower lobe as the initial plan. After division of the pulmonary artery and the lower lobe bronchus and dissection of the fissures between upper and middle lobes with staplers, intrapericardial dissection of the slit-like pulmonary vein was performed with a stapler. Then, finger dissection between pericardium and tumor was performed. The dissection between left atrium and tumor was laborious and undertaken carefully using fingers, electrocautery, and a vessel-sealing instrument. At the final stage of dissection, the left atrial wall was lacerated. Once, we successfully controlled bleeding by using a hemostat in the left atrial laceration part. Therefore, we continued the dissection between left atrium and tumor. The tumor finally dissected free from the left atrium. After complete removal of the specimen (Figure 3) from the surgical field, the hemostat slipped, extensive bleeding then occurred, and the blood pressure decreased, resulting in circulatory arrest. We repaired the 2.5 cm laceration of the atrial wall with go and back continuous suture using 3-0 Prolene during cardiac arrest. After air removal from the atrium, polyglycolic acid sheet with fibrin glue was pasted over the suture line. Then, direct cardiac massage was performed, the anesthesiologist continued 100% oxygen support, and platelet-rich plasma and 1 g of hydrocortisone were intravenously administered. Subsequently, her heartbeat returned and blood circulation was restored. Total ischemic time was 10 minutes. After securing hemostasis completely, a 24 Fr chest tube was placed and the chest was then closed. Intraoperative blood loss was estimated at 5200 mL. The operation time was 270 minutes. After the operation, the patient was hemodynamically stable and transferred to the intensive care unit.

Her postoperative course was uneventful. She was neurologically intact and successfully extubated on postoperative day 1 (POD 1). The chest tube was removed on POD 4. She was discharged with no complications on POD 14. To date, she has shown no evidence of recurrence for 15 months.

## 3. Discussion

Mediastinal paragangliomas are rare tumors, representing less than 1% of all mediastinal tumors and less than 2% of all pheochromocytomas. Functioning paragangliomas are often discovered by the symptoms of hypertension, headache, sweat, and tremor secondary to catecholamine secretion. However, nonfunctioning paragangliomas are asymptomatic and often found incidentally, sometimes after the formation of a large mass, as in our presented case. Because of firm adhesion of the tumor to both pericardium and atria, we were not able to identify the exact site of origin. We suppose that this tumor might have originated from parasymptomatic-related tissue in the pericardium or atria.

Paragangliomas have typical imaging characteristics on CT and MRI scans. Enhanced CT scans of paragangliomas usually demonstrate a heterogeneous mass with peripheral enhancement and sometimes central areas of low attenuation, most likely representing tumor degeneration or necrosis. MRI shows intermediate signal intensity on T1-weighted images and high signal intensity on T2-weighted images [2]. ^123^I-metaiodobenzylguanidine (^123^I-MIBG) scintigraphy remains important in pheochromocytoma and functioning paraganglioma [3]. The more functional the tumor is, the higher the MIBG accumulation is and the easier the scintigraphic detectability is. Our case was normotensive and revealed higher uptake of FDG on PET imaging. Thus we suspected a malignant mesenchymal tumor such as high-grade sarcoma or lymphoma, and preoperative tissue diagnosis was indicated.

According to a literature review by Lamy et al. [1], anterior and middle mediastinum paragangliomas are locally invasive and have a high local recurrence rate (55.7%), with true metastatic capacity (26.6%). The survival rate with complete resection is 84.65% versus 50.0% for patients with partial resection and adjuvant therapies. Our case presented a benign histologic appearance. However, there are no reliable morphologic criteria by which to separate the benign from the malignant forms, although high mitotic activity and decreased immunohistochemical reactivity for neuropeptides correlate with clinical malignancy.

Pericardial or cardiac paragangliomas are very rare. The treatment of choice is complete resection. Preoperative embolization to reduce perioperative bleeding may be considered for these hypervascular tumors [4]. Actually, we experienced hemorrhage from the tumor at the time of incisional biopsy. However, since the tumor was surrounded by pseudocapsule, this may have controlled intraoperative bleeding from the tumor at the time of complete resection. On the other hand, at radical surgery, some researchers employed cardiopulmonary bypass [1, 5] in order to avoid the intraoperative catastrophic hemorrhage as we experienced, but others reported a case successfully treated without cardiopulmonary bypass [4, 6]. We considered the need initially to secure a good operative field, namely, by posterolateral thoracotomy, and successfully undertook repair of the atrial laceration under the excellent operative field without cardiopulmonary bypass. This approach and patient's position had an additional advantage of avoiding air embolism, because the lacerated wound was located at the top of the left atrium. We could sew the wound while blood filled the atrium. However, we supposed that cardiopulmonary bypass may be standby if the tumor location is close to great vessels or heart, as in our presented case.

In conclusion, a large intrapericardial paraganglioma was successfully removed with the right lower lobe via posterolateral thoracotomy. In light of our experience and the possibility of publication bias, cardiopulmonary bypass should be considered, if surgeons are able to secure suitable sites for arterial and venous cannulations while right posterolateral thoracotomy is employed. In addition, diligent long term follow-up is mandatory for this tumor with malignant potential.
